# Interventions on Metabolism: Making Antibiotic-Susceptible Bacteria

**DOI:** 10.1128/mBio.01950-17

**Published:** 2017-11-28

**Authors:** Fernando Baquero, José-Luis Martínez

**Affiliations:** a Department of Microbiology, Ramón y Cajal Institute for Health Research (IRYCIS), Ramón y Cajal University Hospital, CIBERESP, Madrid, Spain; b Centro Nacional de Biotecnología, CSIC, Madrid, Spain

**Keywords:** antibiotic resistance, bacterial metabolism, polymyxins, recovering susceptibility

## Abstract

Antibiotics act on bacterial metabolism, and antibiotic resistance involves changes in this metabolism. Interventions on metabolism with drugs might therefore modify drug susceptibility and drug resistance. In their recent article, Martin Vestergaard et al. (mBio 8:e01114-17, 2017, https://doi.org/10.1128/mBio.01114-17) illustrate the possibility of converting intrinsically resistant bacteria into susceptible ones. They reported that inhibition of a central metabolic enzyme, ATP synthase, allows otherwise ineffective polymyxin antibiotics to act on *Staphylococcus aureus*. The study of the intrinsic resistome of bacterial pathogens has shown that several metabolic genes, including multigene transcriptional regulators, contribute to antibiotic resistance. In some cases, these genes only marginally increase antibiotic resistance, but reduced levels of susceptibility might be critical in the evolution or resistance under low antibiotic concentrations or in the clinical response of highly resistant bacteria. Drug interventions on bacterial metabolism might constitute a critical adjuvant therapy in combination with antibiotics to ensure susceptibility of pathogens with intrinsic or acquired antimicrobial resistance.

## COMMENTARY

One of the complications of treating infections is the existence of intrinsically resistant microorganisms. At first glance, the term “intrinsic resistance” implies a clear cutoff. If an organism is intrinsically resistant to a given antibiotic, the drug is useless for treating infections with this microorganism. The recent publication of an article by Martin Vestergaard et al., “Inhibition of the ATP synthase eliminates the intrinsic resistance of *Staphylococcus aureus* towards polymyxins,” together with some other previous works on metabolic interventions, challenges this paradigm (1). From a chemical point of view, an antibiotic is active if it reaches its target at a sufficient concentration for inhibition of target function or activity. Consistent with this view, resistance can then be achieved because of just two situations (or a combination of both): the antibiotic cannot reach enough concentration to act on the target, or the target-antibiotic affinity is naturally low. If the affinity for the target is low, the best strategy to overcome resistance is to develop antibiotics with a better affinity. However, if the problem deals with the concentration of the antibiotic available on-target, metabolic interventions may improve the activity of the antibiotic. Among intervention strategies, the use of coadjuvants of metabolites to induce the internalization of the antibiotic, to inhibit its detoxification or extrusion, to induce endogenous oxidative stress, or to prime to proton motive force, as in the case of persister cells, has been proposed as a useful strategy to resensitize antibiotic-resistant bacteria (2).

In this context, it is relevant to distinguish between intrinsic and acquired resistance. Acquired antibiotic resistance, particularly mutations resulting in a genetic alteration of the drug target itself or the targeted metabolic process, has consequences in bacterial metabolism. There is an erroneous expansion of the Ehrlich’s concept of “magic bullet”: the host metabolic homeostasis might be spared from the action of the magic bullet, but certainly not the metabolism of the target bacterial cell, as any toxic or deleterious (stressful) effect has metabolic consequences. Certainly, the antibiotic-derived bacterial growth inhibition or death effects are, respectively, consequences of metabolic illness, eventually leading to a metabolic catastrophe. Correspondingly, acquired resistance mechanisms can be linked to metabolic adaptations (3), building alternative or mutant routes for the inhibited path, alternative architectures for the inhibited macromolecules, hyperexpressing homeostatic mechanisms, or causing deviations in protein synthesis to produce detoxifying enzymes. Many of these metabolic changes resulting from resistance result in “fitness costs,” expressing how the cellular metabolism has deviated from optimality. However, these changes offer a wealth of novel metabolic targets upon drugs which eventually, devoid of antibiotic activity by themselves, may serve to specifically fight against antibiotic-resistant bacteria.

Intrinsic antibiotic resistance, on the other hand, is the result of the normal metabolic functions of the cell, making the cell naturally unsusceptible to particular antimicrobial agents. Indeed, the study of the intrinsic resistome of bacterial pathogens has shown that several metabolic genes contribute to antibiotic resistance (4). However, we can certainly distort the natural metabolism by drugs that might not necessarily be antibiotics. This “drug-altered metabolism” can eventually render the cell susceptible to certain antimicrobials. A variant of “intrinsic resistance” is “phenotypic resistance,” when a portion of an otherwise antibiotic-susceptible bacterial cell population enters into a drug-refractory type of metabolism, eventually activated by the stationary growth phase, which in its turn is related to changes in the environment. Hypothetically, we can also use drugs to turn this refractory metabolism to one susceptible to antibiotics.

The recent publication by Vestergaard et al. (1) provides an excellent occasion to discuss the possibility of making antibiotic-susceptible bacteria from otherwise intrinsically resistant organisms by modifying the basic cell metabolism. The authors systematically screened a *Staphylococcus aureus* transposon library for polymyxin susceptibility. They found that mutations involving subunits of the ATP synthase, providing a central function in bacterial metabolism, cause the intrinsic polymyxin-resistant phenotype to revert to susceptibility. This result was confirmed by treating the cells with the macrolide oligomycin A, an ATP synthase inhibitor that renders *S. aureus* susceptible to polymyxins.

What could be the mechanism of acquisition of polymyxin susceptibility? Certainly ATP synthase is a “splendid molecular machine” (5), contributing in an essential way to bacterial growth. This molecular machine is composed of several proteins (cotranscribed in a single polycistron), one of which is AtpA; according with Vestergaard et al., transposon mutants of this gene are very effective in allowing polymyxin activity (1). The AtpA protein has a high stoichiometry in the ATP synthase complex and requires a high synthesis rate, depending on the availability of a sufficiently large density of ribosomes (6). However, both the density of ribosomes and the transcription-translation processes are very energy (ATP) demanding (6), and one can envisage a possible conflict between the rate of ATP production and the ATP required for construction of the ATP synthase complex. This situation might critically affect the density of the more demanding proteins in the bacterial cell, such as the ribosomal and cell wall proteins. Data from *Escherichia coli* indicate that dominant among these proteins (nearly 2% of the proteome) are RpsA (30S ribosomal subunit protein S1), influencing ribosome biogenesis, and Lpp (murein lipoprotein) (6). Classical genetic studies have demonstrated that mutants defective in Lpp are hypersusceptible to hydrophobic antibiotics, cationic dyes, and some detergents (7). As much as 16% of all *S. aureus* proteins during aerobic growth are ribosomal proteins (8). Murein lipoproteins, which are also anchored to the outer leaflet of the cytoplasmic membrane and which may extend into the cell wall and beyond, have been detected and account for about 25% of all surface-associated proteins. However, their role in constraining the permeation of molecules as polymyxins has not been evaluated. Regardless, polymyxin susceptibility may be a consequence of an increased “cost of life” under conditions of energetic shortage (8), leading to reduced cell ribosome numbers, known to increase the cellular susceptibility to some antibiotics (9).

The article by Vestergaard et al. focuses on the possibility of increasing the susceptibility of bacteria targeting intrinsic resistance mechanisms, including physiological metabolic alterations influencing susceptibility (1). How relevant are these for gaining therapeutic success? Of course, the case of *Staphylococcus aureus* is an extreme one, as the organism is intrinsically resistant to high polymyxin concentrations. However, we should also target the much more frequent case of “intrinsic mechanisms” to reduce antibiotic susceptibility in already susceptible organisms. In most cases, the elimination of these mechanisms will only result in a 2 to 4× reduction of the MIC, an apparently small gain in the efficacy of the antibiotic. How important it is to achieve this goal?

**FIG 1:**
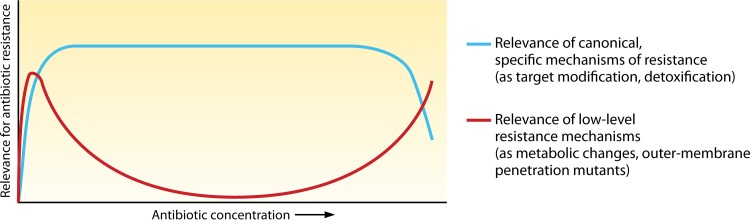
Low-level resistance mechanisms (2 to 4× MIC), including low-level intrinsic resistance, might be relevant in the early evolution of resistance, allowing bacteria to persist in the presence of low antibiotic concentrations and providing them the opportunity of evolving toward more effective resistance. Also, when canonical specific mechanisms of resistance are surpassed by high concentrations of antibiotics, the 2- to 4× MIC increases provided by “low-level mechanisms” might be critical to produce therapeutic failure.

In Fig. 1, we consider that interventions directed to intrinsic low-level resistance mechanisms (2 to 4× MIC) might be of clinical interest. First, consider an absence of specific mechanisms of resistance when the bacteria are exposed to very low antibiotic concentrations. If the wild-type strain has a MIC of 0.01 µg/ml, a 4-fold increase results in a MIC of 0.16, but this small change, allowing survival in areas with low antibiotic concentrations (10), might be critical to avoid the development of acquired resistance by mutation or horizontal gene transfer, and therefore interventions to prevent such a small rise in the MIC could be eventually justified. Second, consider when the specific resistance mechanism is not sufficient to ensure survival at high antibiotic concentrations; for instance, a mere 4× decrease in the MIC moves the MIC of a given antibiotic from a nonachievable concentration at 128 µg/ml to an accessible concentration of 8 µg/ml, making an effective therapy possible.

Interventions to make antibiotic susceptible those pathogens that have spontaneous low-level or high-level intrinsic mechanisms of resistance, and also those that have acquired resistance by mutation or horizontal gene transfer, can probably be achieved with adjuvant drugs altering bacterial metabolism. This approach offers a promising perspective for the therapy of resistant infections in an antibiotic-polluted biosphere.
